# Hierarchical Cascade‐Activated Microneedle Patches for Photothermal and Chemotherapy

**DOI:** 10.1002/smmd.70059

**Published:** 2026-09-30

**Authors:** Gaizhen Kuang, Wenzhao Li, Yu Wang, Qingfei Zhang, Hua Jiang

**Affiliations:** ^1^ Department of Radiation and Medical Oncology Joint Centre of Translational Medicine‐WIUCAS The First Affiliated Hospital of Wenzhou Medical University Wenzhou China; ^2^ School of Basic Medical Sciences Wenzhou Medical University Wenzhou China; ^3^ Department of Otolaryngology The Second Affiliated Hospital Zhejiang University School of Medicine Hangzhou Zhejiang China

**Keywords:** bioorthogonal reaction, cancer, chemotherapy, microfluidics, microneedle

## Abstract

Chemotherapy and photothermal therapy (PTT) are recognized as effective strategies in cancer treatment. However, the challenge remains in effectively targeting these therapeutic modalities to the tumor site to improve therapeutic effects. Here, a novel hierarchical cascade‐activated microneedle (MN) patch (MN_DOX_@M_ICG_) for photothermal and bioorthogonal chemotherapy was developed. The MN patch was fabricated by integrating hydrogel microspheres (M_ICG_) containing tetrazine‐functionalized indocyanine green (Tz‐ICG) into the MN arrays. Upon irradiation with near‐infrared light, Tz‐ICG with high photothermal conversion efficiency can elevate the temperature of MN, facilitate PTT, and trigger the release of *trans*‐cyclooctene‐conjugated doxorubicin (TCO‐DOX) encapsulated in MN. Then, the gradually released Tz‐ICG from M_ICG_ can react with TCO‐DOX via a specific inverse electron‐demand Diels‐Alder bioorthogonal reaction, thereby restoring the anticancer activity of DOX. Consequently, MN_DOX_@M_ICG_ can efficiently eradicate tumor cells in vitro and significantly inhibit the growth of postsurgical melanoma by combining PTT with bioorthogonal chemotherapy. These findings highlight the practical value of such a hierarchical cascade‐activated MN patch for versatile drug delivery and biomedical applications.

## Highlights

- Hierarchical cascade‐activated microneedle patches (MN_DOX_@M_ICG_) are developed by integrating microfluidic hydrogel microspheres into microneedle arrays.
- The MN_DOX_@M_ICG_ loaded with TCO‐DOX and Tz‐ICG showed efficient photothermal therapy performance and bioorthogonal chemotherapy ability.
- The microneedle system significantly inhibits postsurgical melanoma growth, providing an effective strategy for cancer combination therapy.

## Graphical abstract

Hierarchical cascade‐activated microneedle patches (MN_DOX_@M_ICG_) were developed by integrating microfluidic hydrogel microspheres into microneedle arrays, enabling precise integration of photothermal therapy (PTT) and bioorthogonal chemotherapy. Under near‐infrared irradiation, the MN_DOX_@M_ICG_ showed efficient Tz‐ICG‐mediated photothermal performance and triggered a bioorthogonal reaction to restore DOX's anticancer activity for synergistic tumor suppression.

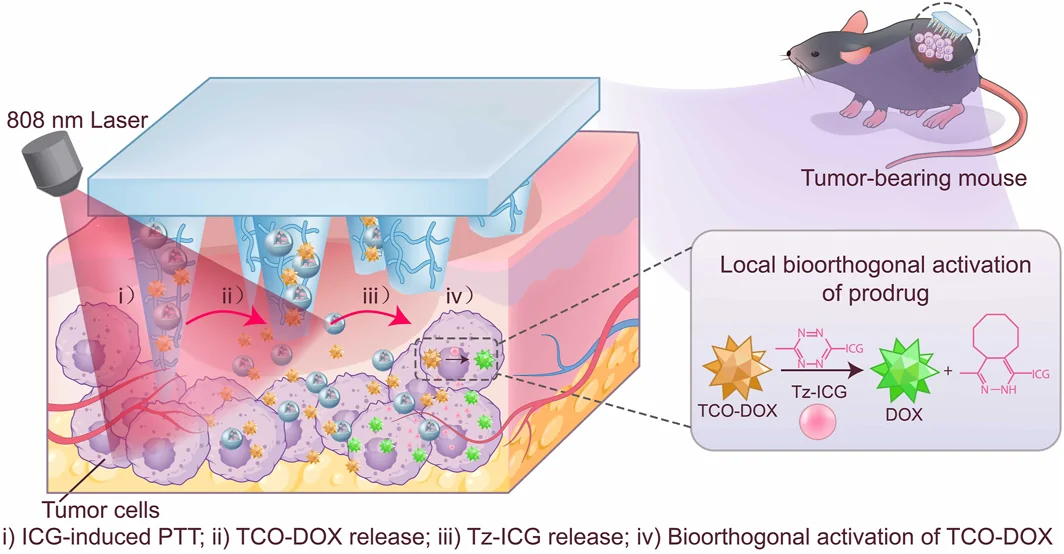

## 1 Introduction

Cancer represents a prominent worldwide public health problem, significantly affecting human survival and overall well‐being [1]. Chemotherapy, regarded as one of the primary conventional treatment modalities, has long been an indispensable part of cancer therapy [2, 3]. Specifically, the strategy of activating prodrugs through bioorthogonal reactions for chemotherapy, such as doxorubicin (DOX) prodrugs, has achieved promising therapeutic effects [4, 5, 6]. However, the restricted bioavailability and administration vulnerability of prodrugs lead to inadequate drug levels at the tumor, diminishing therapeutic effectiveness [7, 8]. Besides, adverse effects, drug resistance, and tumor complexity result in unsatisfactory therapeutic outcomes of chemotherapy [9, 10]. In comparison, photothermal therapy (PTT) has gained substantial interest due to its low invasiveness, strong spatiotemporal specificity, and reliable controllability [11, 12, 13, 14]. To attain enhanced antitumor efficacy, a variety of drug delivery vehicles (including films, hydrogels, fibers, and microspheres) have been engineered through the combination of chemotherapy and PTT [15, 16, 17, 18, 19]. Unfortunately, the combination delivery systems commonly encounter several challenges that hinder their broader application. The straightforward integration of drugs/photosensitizers and carrier materials often leads to uncontrollable release behavior [20]. In addition, the unavoidable release of toxic therapeutic drugs or photosensitizers from these delivery carriers can cause significant harm to healthy tissues [21, 22]. Therefore, the development of a smart system that integrates intelligent drug activation with innovative therapeutic modalities holds great promise for tumor therapy.

In this paper, we report a novel hierarchical cascade activated microneedle (MN) patch for photothermal and chemotherapy of melanoma, as schemed in Figure 1. MN patch, as a minimally invasive transdermal drug delivery strategy, has advantages such as high drug loading capacity, good biocompatibility, painlessness, minimal damage, self‐management, simple handling, and wide application [23, 24, 25]. As a promising local drug delivery system, MN patches can effectively load therapeutic reagents such as chemical drugs, nanomedicines, proteins, and nucleic acids, achieving controllable and continuous release to meet the treatment needs of various diseases [26, 27, 28]. Based on these characteristics and advantages, the MN patch system can achieve more ideal tumor treatment effects compared with traditional drug delivery methods [29, 30]. Specifically, research has fabricated a bioorthogonal catalytic MN patch that facilitates the localized activation of doxorubicin (DOX) prodrug at subcutaneous melanoma sites, thereby restoring the therapeutic anticancer efficacy of the parent drug [31]. Although it achieved certain treatment effects, the system lacks a well‐defined structural design and combination therapy potential, resulting in the substandard anticancer performance. Therefore, it is conceived that by elaborating a design, a multifunctional MN therapeutic platform could be developed to improve tumor therapy efficiently.

**FIGURE 1:**
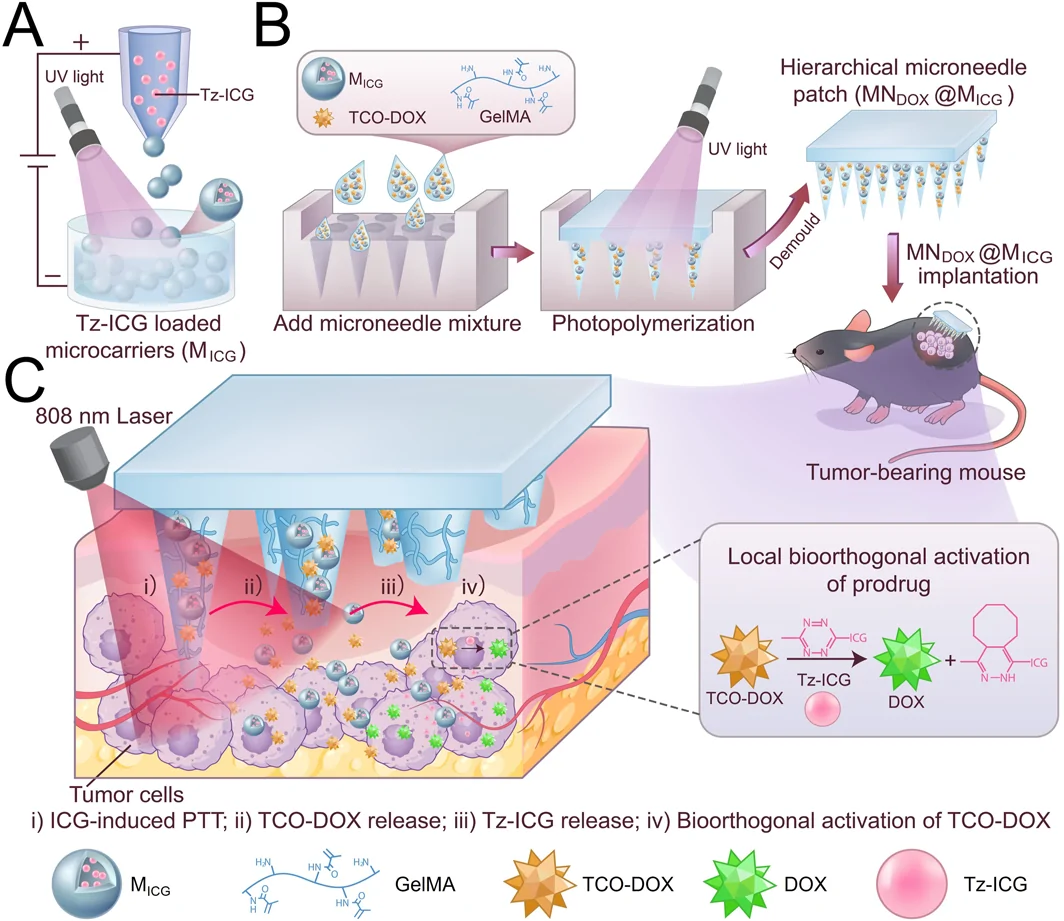
Hierarchical microneedle patch (MN_DOX_@M_ICG_) for tumor therapy. (A) Fabrication of M_ICG_ with Tz‐ICG loading using the microfluidic electrospray technique. (B) Fabrication of MN_DOX_@M_ICG_. (C) The therapeutic processes and mechanisms of the MN_DOX_@M_ICG_.

Herein, we combined the microfluidic hydrogel microspheres and the MN arrays together to present a novel microsphere‐integrated hierarchical cascade activated MN patch (MN_DOX_@M_ICG_) for controllable bioorthogonal prodrug activation and combination therapy (Figure 1). The hydrogel microspheres (M_ICG_), containing tetrazine‐functionalized indocyanine green (Tz‐ICG), were generated by a flexible capillary microfluidic electrospray method [32, 33]. These M_ICG_ were embedded into the MN arrays containing *trans*‐cyclooctene‐conjugated doxorubicin (TCO‐DOX). Compared to currently existing drug delivery systems, the design of microspheres in MN facilitates the separate loading of bioorthogonal prodrug molecules. This approach prevents premature activation of the prodrug molecules and achieves a gradual drug release behavior, thereby enhancing the localized delivery and action of the drug. As a kind of biocompatible near‐infrared (NIR) absorption material, the Tz‐ICG has a high photothermal conversion efficiency, which could induce the PTT ability of the MN_DOX_@M_ICG_. Besides, the photothermal effect can accelerate the release of encapsulated TCO‐DOX and Tz‐ICG, thereby facilitating the specific inverse electron‐demand Diels–Alder (IEDDA) reaction. As a typical bioorthogonal reaction, IEDDA can occur without the need of metal catalysts [34, 35]. This reaction utilizes tetrazine moieties to undergo cycloaddition with highly strained cycloalkenes, presenting significant potential for various biomedical applications. Through this bioorthogonal reaction, Tz‐ICG successfully activated TCO‐DOX, thereby restoring the anti‐tumor activity of DOX. Based on these features, MN_DOX_@M_ICG_ exhibited effective in vitro tumor cell cytotoxicity and significantly inhibited post‐surgical melanoma growth by combining PTT and bioorthogonal chemotherapy, accompanied by good safety. These results emphasize that such a hierarchically cascade‐activated MN patch represents an ideal candidate for controllable drug delivery, providing an effective and versatile therapeutic strategy for diverse biomedical applications.

## 2 Results and Discussion

The study employed a glass capillary microfluidic electrospray platform to fabricate Tz‐ICG encapsulated microspheres (illustrated in Figure 2A and Supporting Information S1: Figure S1). The initial steps involved introducing a precursor mixture of polyethylene glycol diacrylate (PEGDA), Tz‐ICG compounds, and a photopolymerization initiator into the microfluidic setup. Within this setup, electrostatic and gravitational forces facilitate droplet formation. Subsequently, the generated droplets were rapidly frozen in a liquid nitrogen bath before undergoing ultraviolet irradiation for polymerization, resulting in the final M_ICG_ formulation. As a control group, empty microspheres (M_B_) lacking therapeutic agents were also manufactured simultaneously. Morphological assessments conducted via confocal laser scanning microscopy (CLSM) and scanning electron microscopy (SEM) demonstrated that both M_B_ and M_ICG_ maintained structural uniformity, displaying spherical geometries with diameter measurements of 150.8 ± 19.4 μm and 151.0 ± 19.1 μm, respectively (Figure 2B–D). Due to the incorporation of Tz‐ICG, the M_ICG_ exhibited intense red fluorescence under excitation light as observed through CLSM, whereas this fluorescence was absent in the M_B_ (Figure 2B). Besides, the M_ICG_ appeared green in the tubes, in contrast to the M_B_, which appeared white (Supporting Information S1: Figure S2). The spherical morphology of the microspheres was examined through SEM, revealing a significant reduction in the average diameter subsequent to the dehydration process (Figure 2E).

**FIGURE 2:**
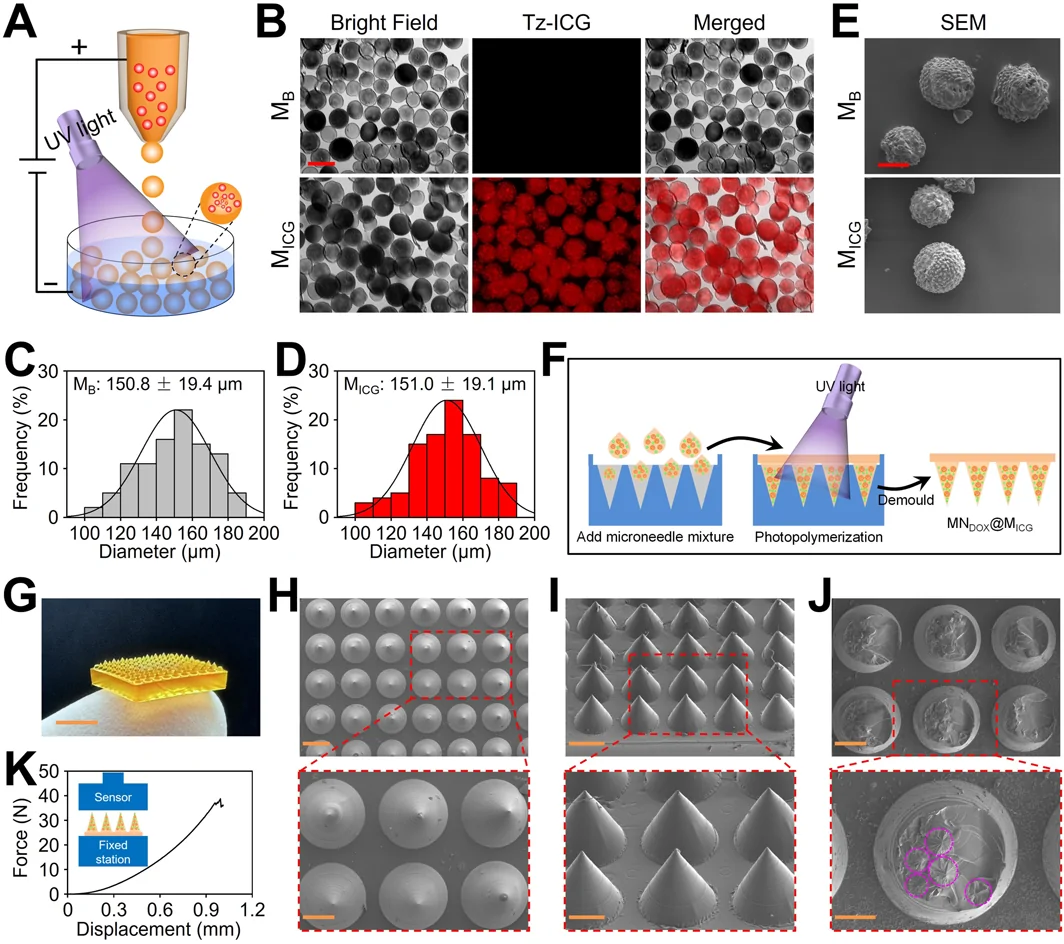
Preparation and characterization of MN_DOX_@M_ICG_. (A) Fabrication processes of the microspheres. (B) Bright‐field and fluorescence microscopy images of M_B_ and M_ICG_. Scale bar: 200 μm. (C, D) Size distributions of M_B_ and M_ICG_. (E) SEM images of M_B_ and M_ICG_. Scale bar: 50 μm. (F) Fabrication procedure of MN_DOX_@M_ICG_. (G) Photograph of MN_DOX_@M_ICG._ Scale bar: 5 mm. (H, I) The top and lateral views of the MN_DOX_@M_ICG_. Scale bars: 500 μm for the upper image and 250 μm for the lower image. (J) Cross‐sectional SEM images of the MN_DOX_@M_ICG._ Scale bars: 250 μm for the upper image and 150 μm for the lower image. (K) The compressive force–displacement curve of the MN_DOX_@M_ICG_.

The hierarchical MN patches were manufactured through a template replication methodology [27, 36]. Figure 2F illustrates the preparation processes, where a homogeneous mixture of methacrylate gelatin (GelMA) and TCO‐DOX was combined with microspheres before being precisely deposited into the tip regions of a negative mold. Subsequently, an additional mixture of GelMA was applied to form the backing layer within the mold. It should be noted that the TCO‐DOX conjugate was prepared following established synthetic protocols [37, 38], with its successful formation confirmed by characteristic ^1^H NMR signals shown in Supporting Information S1: Figures S3 and S4. Upon UV exposure, both the tip and backing components underwent complete photopolymerization and curing. The final hierarchical MN patch was then obtained through demolding after UV‐induced crosslinking. As shown in Figure 2G, the fabricated device exhibited a well‐defined architecture comprising a 10 × 10 array of conical microneedles mounted on a 12 × 12 mm^2^ backing substrate. Individual needles featured geometric parameters of 960 μm base diameter and 800 μm length. SEM analysis further revealed the highly ordered spatial distribution of MN across the backing layer (Figure 2H,I), while cross‐sectional examination clearly visualized the embedded microspheres (Figure 2J). Mechanical characterization indicated each needle could withstand compressive loads up to 0.38 N (Figure 2K), collectively demonstrating the successful production of structurally intact and mechanically robust hierarchical MN patches. For the convenience of explanation, the MN patches are named as follows: MN_B_@M_B_ for those with M_B_ but without loading TCO‐DOX, MN_B_@M_ICG_ for those with M_ICG_ but without loading TCO‐DOX, MN_DOX_@M_B_ for those with M_B_ and TCO‐DOX, and MN_DOX_@M_ICG_ for those with both M_ICG_ and TCO‐DOX.

As a widely employed photothermal therapeutic agent, ICG serves as the foundational compound for developing Tz‐ICG derivatives [39, 40]. Given the structural homology among these compounds, we hypothesized that Tz‐ICG would exhibit similar photothermal conversion properties. This hypothesis led to a systematic investigation of the thermal response profiles demonstrated by the MN system. As shown in Figure 3A, under 808 nm laser exposure, both MN_B_@M_ICG_ and MN_DOX_@M_ICG_ achieving temperature elevation of Δ29.5°C and Δ30.0°C, respectively, within a 3‐min irradiation period. MN_B_@M_B_ and MN_DOX_@M_B_, lacking M_ICG_ components, showed minimal thermal response under the same experimental conditions. Besides, the photothermal temperature changes of the MN_DOX_@M_ICG_ were significantly increased with the increase in the laser power density (Figure 3B). Remarkably, the MN system maintained reproducible thermal output through five consecutive NIR irradiation cycles (Figure 3C), demonstrating the exceptional photostability of the system. Infrared thermographic analysis provided spatial‐temporal visualization of the photothermal activation dynamics across the MN arrays (Figure 3D). These experimental findings collectively verified the superior photothermal performance of MN platforms incorporating Tz‐ICG, thereby providing a promising technological basis for the development of photothermally mediated therapeutic interventions.

**FIGURE 3:**
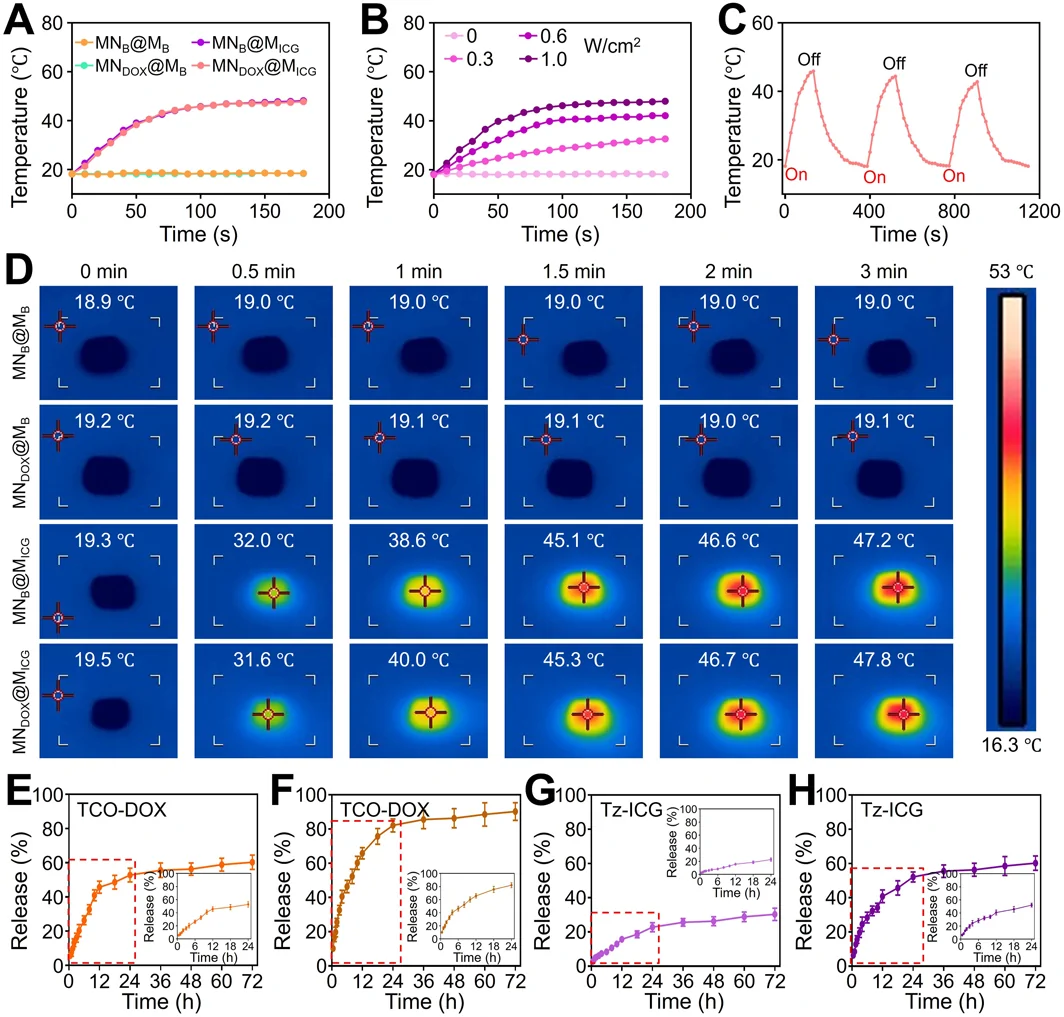
Photothermal effects and in vitro drug release of MN_DOX_@M_ICG_. (A) Thermal response profiles of various MN patches under 808‐nm laser exposure (0.6 W cm^−2^). (B) Power‐dependent photothermal changes of MN_DOX_@M_ICG_. (C) Thermal stability testing of MN_DOX_@M_ICG_ through three On/Off cycles (0.6 W cm^−2^, 100 s irradiation followed by 300 s recovery intervals). (D) Thermal imaging photographs of different MN under irradiation (0.6 W cm^−2^). (E) TCO‐DOX release profile without irradiation. (F) TCO‐DOX release profile with irradiation. (G) Tz‐ICG release profile without irradiation. (H) Tz‐ICG release profile with irradiation.

The absorption spectra of TCO‐DOX and Tz‐ICG were first detected before measuring the release profiles of the hierarchical MN system (Supporting Information S1: Figure S5). Subsequently, a quantitative analysis of the drug‐release profiles of the system was conducted utilizing UV‐vis‐NIR spectroscopy. The TCO‐DOX encapsulated within the MN structures exhibited relatively rapid release kinetics in the absence of irradiation, achieving a cumulative release of 52.8% within a 24‐h observation period (Figure 3E). Over the next 48 h, the release amount gradually increased to 60.3% at 72 h. Upon exposure to irradiation, the release rate of TCO‐DOX was significantly enhanced, with the release amount rapidly reaching 82.1% within 24 h and ultimately 90.2% at 72 h (Figure 3F). In contrast, the Tz‐ICG loaded in the M_ICG_ displayed much slower release characteristics, requiring extended duration to achieve comparable release percentages, regardless of irradiation (Figure 3G,H). This differential release behavior highlights the capacity of such hierarchical MN system for sequential drug release, a feature that could contribute to controlled drug activation and prolonged anticancer therapeutic applications.

The anticancer effect of MN_DOX_@M_ICG_ was evaluated through CCK‐8 proliferation assay and live/dead staining assay. Primary investigations focused on the cytotoxic profile change of TCO‐DOX by comparing its bioactivity pre‐ and post‐activation by Tz‐ICG. Quantitative cytotoxicity analysis revealed distinct differences in therapeutic potency between free DOX and TCO‐DOX. Specifically, free DOX exhibited an IC_50_ value of 0.39 μM, whereas the TCO‐conjugated prodrug showed markedly attenuated cytotoxicity with an IC_50_ of 10.5 μM (Figure 4A). This significant reduction in cytotoxicity is attributed to the covalent bonding between the cyclooctene moiety and the amino group at the anticancer active site of DOX [38]. Subsequent bioorthogonal activation through the IEDDA reaction with Tz‐ICG restored therapeutic efficacy (Supporting Information S1: Figure S6), decreasing the IC_50_ to 0.48 μM, comparable to that of free DOX. Control experiments demonstrated the negligible cytotoxicity of Tz‐ICG (Supporting Information S1: Figure S7), thereby confirming the biocompatibility of the reaction. Next, a transwell co‐culture system with B16F10 melanoma cells seeded in lower compartments and MN_DOX_@M_ICG_ applied to upper compartments was used to investigate the anticancer effect of the MN systems (Figure 4B). After a 5‐min photoirradiation followed by a 24‐h incubation period, CCK‐8 assays indicated treatment‐specific responses. The MN_B_@M_B_ and MN_B_@M_ICG_ treatment groups showed minimal proliferative interference, whereas MN_DOX_@M_B_ induced moderate growth suppression (Figure 4C). Notably, non‐irradiated MN_DOX_@M_ICG_ demonstrated superior cytotoxic efficacy compared to MN_DOX_@M_B_, while photothermally activated MN_B_@M_ICG_ (+) exhibited enhanced anticancer ability relative to its non‐irradiated form. The most significant anticancer effects were observed in the MN_DOX_@M_ICG_ (+) treatment group by the combination of bioorthogonal chemotherapy and PTT. These quantitative findings were also corroborated by the live/dead staining assay (Figure 4D), demonstrating the superior anticancer performance of the dual‐modality therapeutic platform of MN_DOX_@M_ICG_.

**FIGURE 4:**
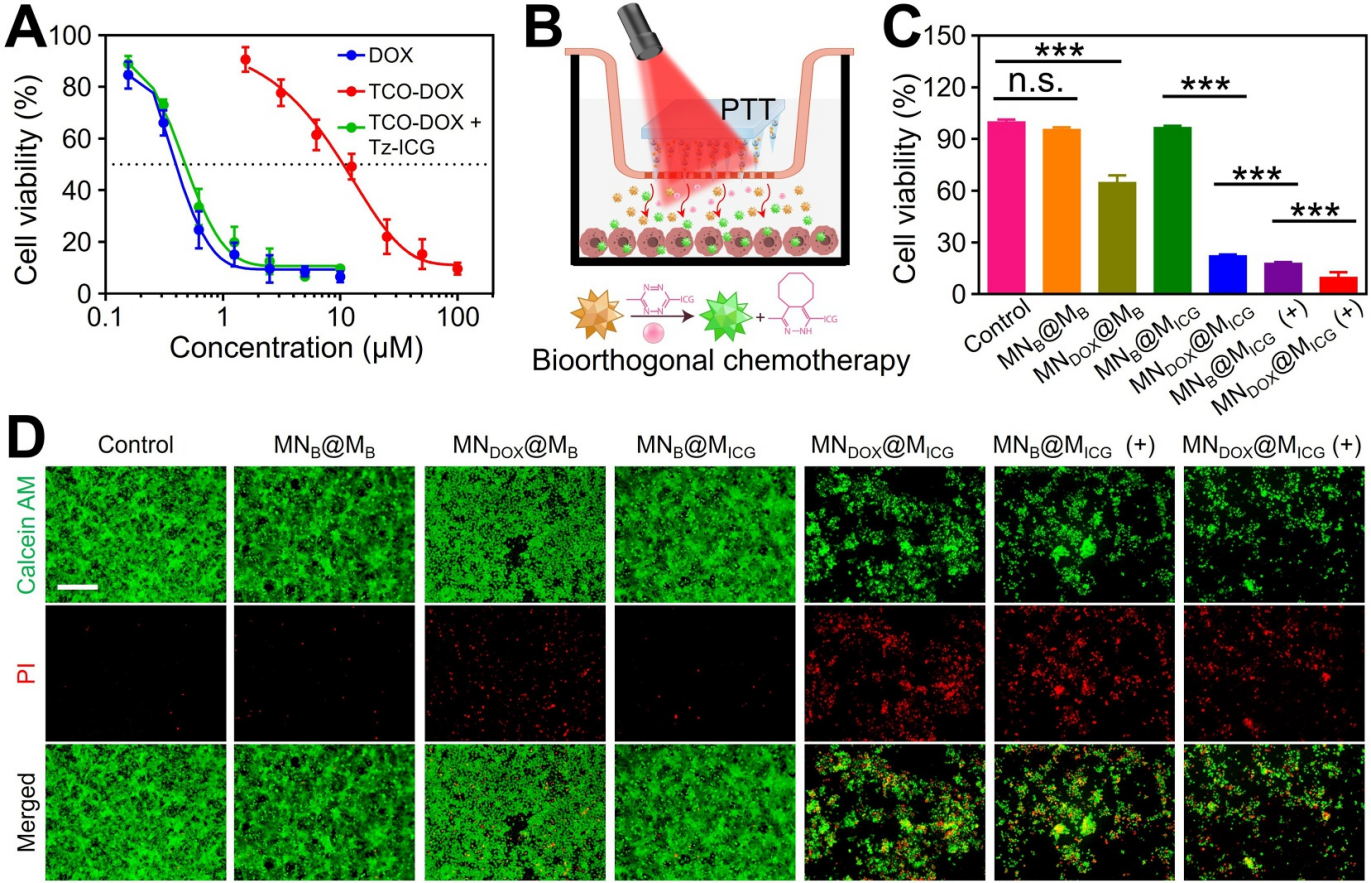
In vitro antitumor efficacy of MN_DOX_@M_ICG_. (A) Cell viability of B16F10 cells after treatment with different drugs. (B) Schematic illustration of the therapeutic processes of MN_DOX_@M_ICG_ against B16F10 melanoma cells. (C) Cell viability of B16F10 cells after treatment with different MN patches, with or without NIR light irradiation (808 nm, 0.6 W cm^−2^, 5 min). (D) Live/dead staining images of B16F10 after treatment with various MN patches, with or without NIR light irradiation (Scale bar: 100 μm). (+) indicated with irradiation. All the cell experiments had three independent replicates (*n* = 3). Data are presented as the mean ± SD. n.s., no significance, *p < 0.05, **p < 0.01, ***p < 0.001.

The in vivo therapeutic efficacy of MN_DOX_@M_ICG_ was then investigated in detail in a subcutaneous melanoma model. Figure 5A illustrates the experimental treatment protocol. The photothermal performance of the MN system was then evaluated. As shown in Figure 5B, the tumor sites treated with MN_B_@M_ICG_ or MN_DOX_@M_ICG_ exhibited rapid temperature elevation within 5 minutes of NIR irradiation, demonstrating effective photothermal ability. In contrast, the treatment groups of MN_B_@M_B_ and MN_DOX_@M_B_ showed minimal temperature variation at irradiated tumor sites. After that, the monitoring of tumor progression demonstrated the unimpeded growth in both saline‐treated and MN_B_@M_ICG_‐treated groups, attributable to the unlimited proliferation of B16F10 cells (Figure 5C). Comparative efficacy analysis demonstrated that while free DOX and MN_DOX_@M_B_ resulted in modest tumor suppression, the MN_DOX_@M_ICG_ treatment displayed significantly superior outcomes. This enhanced efficacy is attributed to the sequential release of the Tz‐ICG and TCO‐DOX components, followed by their bioorthogonal activation, which amplifies the chemotherapeutic effects. Importantly, PTT mediated by MN_B_@M_ICG_ alone also exhibited measurable antitumor activity. The most substantial therapeutic response was observed with the treatment of MN_DOX_@M_ICG_ with NIR irradiation, leading to the maximal inhibition of tumor growth (Figure 5D,E). Collectively, these findings indicate that the hierarchical MN platform, through its integration of PTT and bioorthogonal chemotherapy, constitutes a highly effective multimodal strategy for cancer treatment.

**FIGURE 5:**
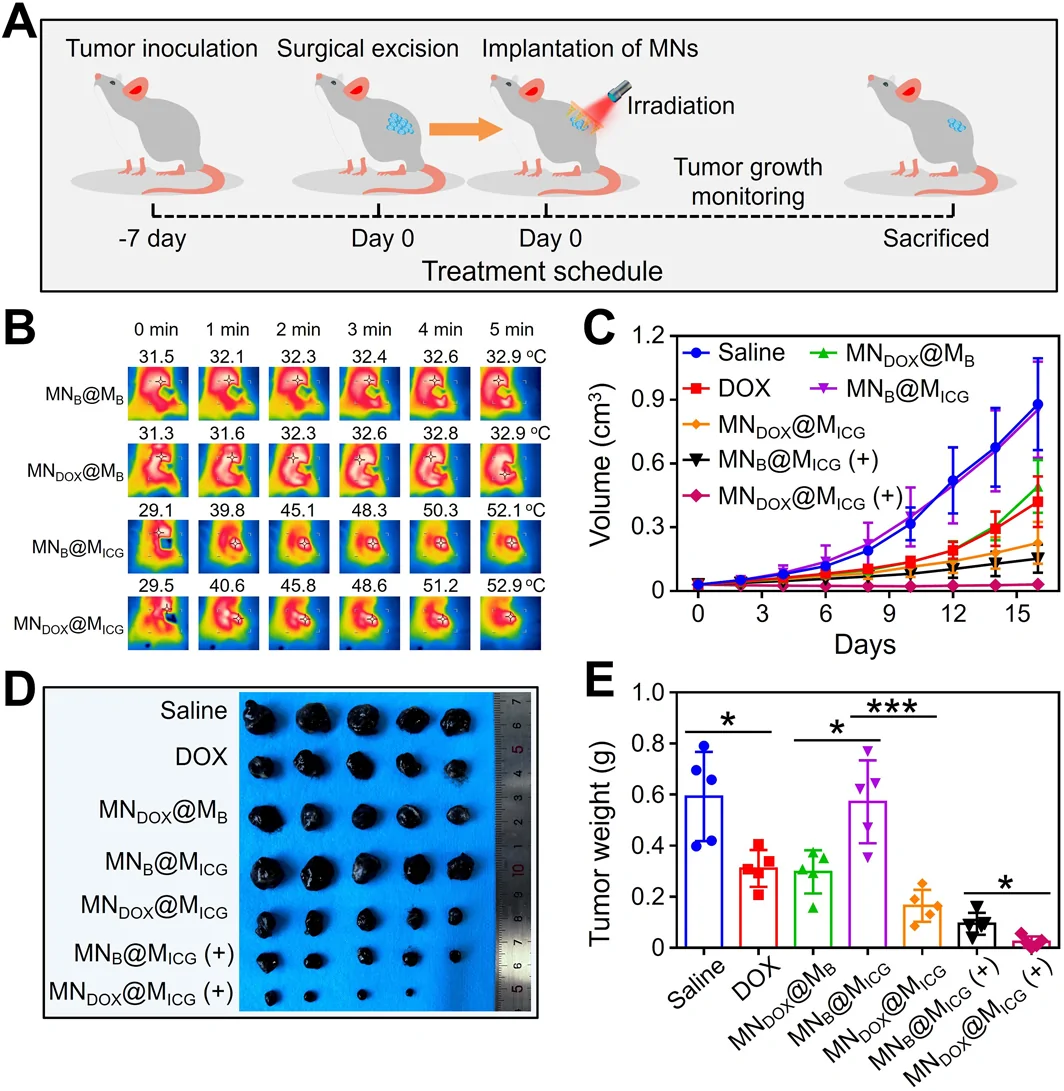
In vivo anti‐tumor performance of MN_DOX_@M_ICG_. (A) The treatment processes of MN_DOX_@M_ICG_. (B) Infrared thermal photographs of mice treated with different MN patches and with NIR irradiation for 5 min (0.6 W cm^−2^). (C) Tumor volume changes of mice after treatment with different MN patches. (D, E) Tumor photograph (D) and tumor weight analysis (E) of excised B16F10 tumors at the end of the experiment. Each experimental group had 5 mice (*n* = 5). Data are presented as the mean ± SD. n.s., no significance, *p < 0.05, **p < 0.01, ***p < 0.001.

To elucidate the therapeutic mechanism of the MN system, comprehensive histological evaluations were performed utilizing hematoxylin‐eosin (H&E) staining, terminal‐deoxynucleotidyl transferase‐mediated nick end labeling (TUNEL) apoptosis assays, and Ki67 analysis. Histopathological assessment via H&E revealed densely populated tumor cell populations with intact nuclear morphology in both untreated controls and the non‐irradiated MN_B_@M_ICG_ group (Figure 6A). In contrast, the MN_DOX_@M_ICG_ (+) intervention group exhibited pronounced tumor cell destruction relative to the other treatment groups. Complementary TUNEL analysis also confirmed maximal apoptotic induction specifically in MN_DOX_@M_ICG_ (+)‐treated group (Figure 6B), validating the synergistic potential of combined PTT and bioorthogonal chemotherapy. Ki67 immunostaining further established that MN_DOX_@M_ICG_ (+) administration correlated with the most substantial suppression of tumor cell proliferation activity (Figure 6C). Notably, during the experimental processes, all mice remained alive, and their body weights gradually increased (Supporting Information S1: Figure S8). However, it is important to note that treated with DOX experienced a reduction in weight due to treatment‐associated systemic toxicity. Despite this, the histological integrity of major organ systems was preserved across all therapeutic regimens (Figure 6D), thereby collectively affirming the favorable biosafety profile of the hierarchical MN platform.

**FIGURE 6:**
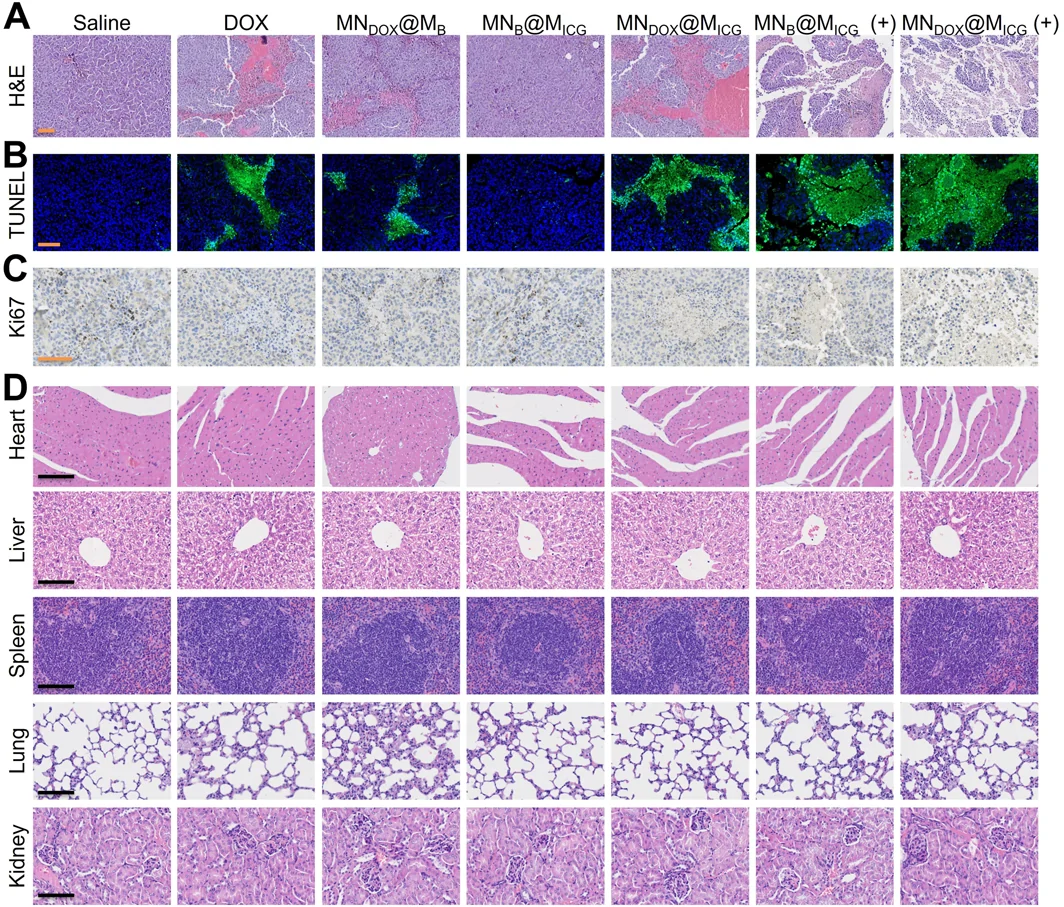
Immunofluorescence and immunohistochemistry analyses. (A–C) H&E, TUNEL, and Ki67 staining analyses of tumors after different treatments (*n* = 5). (D) H&E staining analyses of major organs after different treatments (*n* = 5). Scale bars: 100 μm.

## 3 Conclusion

In summary, this study presents an engineered hierarchical cascade activated MN platform with spatiotemporal control over bioorthogonal reaction agents for combinatorial melanoma intervention. The system design loaded TCO‐DOX within MN needles and Tz‐ICG in microspheres, enabling temporally controlled drug release in response to NIR irradiation. Upon tumor application, M_ICG_‐encapsulated Tz‐ICG demonstrated robust photothermal conversion efficiency under NIR exposure for PTT. Notably, the rapidly released TCO‐DOX from the MN needles underwent sequential activation via an IEDDA reaction with Tz‐ICG released from M_ICG_, effectively restoring the chemotherapeutic efficacy of DOX. Thus, the fabricated MN_DOX_@M_ICG_ platform exhibited pronounced anticancer activity, achieving significant tumor growth suppression through the synergistic effects of photothermal ablation and bioorthogonal chemotherapy, while maintaining exceptional systemic safety. Collectively, this therapeutic platform establishes a novel paradigm for spatially defined drug activation and delivery, demonstrating considerable translational potential in precision tumor therapy.

## 4 Methods

### 4.1 Materials

Tz‐ICG and (E)‐Cyclooct‐2‐en‐1‐yl (4‐nitrophenyl) carbonate ((2E)‐TCO‐PNB) were bought from Confluore Biotechnology Co. Ltd. Poly(ethylene glycol) diacrylate (PEGDA), doxorubicin hydrochloride (DOX·HCl), and the photoinitiator lithium phenyl(2,4,6‐trimethylbenzoyl) phosphinate (LAP) were commercially bought from Aladdin Biochemical Technology. The Calcein‐AM/propidium iodide (PI) dual‐staining kit was obtained from Meilun Biotechnology Co. Ltd. The Cell Counting Kit‐8 (CCK‐8) proliferation assay system was bought from Beyotime Institute of Biotechnology.

### 4.2 Synthesis of TCO‐DOX

The synthesis of TCO‐DOX was according to the previous reports [38]. The ^1^H NMR spectra of (2E)‐TCO‐PNB, DOX, and TCO‐DOX are shown in Supporting Information S1: Figures S3 and S4, which indicate the successful preparation of TCO‐DOX.

### 4.3 Preparation of M_ICG_

The precursor solution containing PEGDA (5% (w/w)), Tz‐ICG (10 mg/mL), and LAP photoinitiator (1% (v/v)) was injected into a glass capillary‐based microfluidic system with an 80 μm orifice diameter. Experimental parameters were precisely controlled, with the syringe pump operating at a flow rate of 1 mL/h and the electrostatic generator maintaining 4 kV output. Subsequently, the droplets generated under the high‐voltage electric field were collected into liquid nitrogen and further cross‐linked by UV light to obtain the M_ICG_. For comparative analysis, blank microspheres (M_B_) were prepared following the same procedures excluding drug incorporation. Structural characterization of both M_B_ and M_ICG_ was performed using optical microscopy and SEM.

### 4.4 Fabrication of MN_DOX_@M_ICG_

The fabrication process began with the preparation of a composite hydrogel solution containing 10% (w/w) GelMA, M_ICG_, 1 mg/mL TCO‐DOX, and LAP. This precursor formulation was introduced into MN‐negative molds, followed by centrifugal filling of the conical microcavities. Subsequently, a secondary coating solution comprising 10% GelMA and 1% LAP was applied to encapsulate the MN backing. Photopolymerization was achieved through 30‐s UV irradiation, resulting in solidification of the MN_DOX_@M_ICG_ construct. The fully cross‐linked MN array was then delicately demolded. For experimental control, three variant MN formulations were prepared: MN_B_@M_B_, MN_B_@M_ICG_, and MN_DOX_@M_B_. Structural evaluation of all MN patches was conducted using both optical microscopy and SEM.

### 4.5 The Photothermal Effect of MN Patches

The thermal response characteristics of the MN arrays under NIR irradiation were systematically examined using an 808 nm wavelength laser system. Quantitative thermal profiling was performed by recording the temporal temperature variations across different MN formulations with an infrared thermographic camera. The influence of irradiation parameters on photothermal conversion efficiency was determined through controlled modulation of laser output power. To evaluate the photothermal stability, the MN system was subjected to three “On‐Off” cycles. Each cycle comprised 100 s of continuous laser irradiation, followed by 300 s of natural cooling under non‐irradiating conditions.

### 4.6 Drug Release Study

Using a UV‐Vis‐NIR spectrophotometric system, the absorption spectra of TCO‐DOX and Tz‐ICG were recorded (Supporting Information S1: Figure S5). Then, to facilitate testing and operation, the release kinetics of Tz‐ICG and TCO‐DOX were investigated from MN_B_@M_ICG_ and MN_DOX_@M_B_, respectively. For TCO‐DOX measurement, the MN_DOX_@M_B_ was placed in a 50 mL centrifuge tube containing 20 mL of PBS. Then, the tube was subjected to controlled oscillation (100 rpm) in a thermostatic chamber maintained at physiological temperature (37°C). At the predetermined time point, 1 mL of release medium was extracted for spectrophotometric quantification of released TCO‐DOX. Equal volume of PBS was replenished to maintain a constant volume. Parallel procedures were administered for MN_B_@M_ICG_ systems, where Tz‐ICG release was monitored at predetermined intervals using the same analytical methodology.

### 4.7 In Vitro Antitumor Study

The experimental protocol began with the seeding of B16F10 melanoma cells at a density of 5 × 10^4^ cells per well in the down chambers of 24‐well transwell plates, allowing 12 h for cellular adhesion. After that, diverse MN formulations were administered into the top chambers. Experimental groups receiving MN_B_@M_ICG_ or MN_DOX_@M_ICG_ interventions were exposed to NIR laser irradiation for 5 min before 24‐h culture incubation, whereas control groups remained in dark condition throughout the incubation period. To quantitatively evaluate the cytotoxic efficacy of MN formulations against tumor cells, parallel assessments were conducted using Calcein‐AM/PI dual staining assay and CCK‐8 assay.

### 4.8 Tumor Inhibition In Vivo

The experimental protocol was initiated with subcutaneous implantation of 1 × 10^6^ B16F10 melanoma cells to the right flank of the mice. Then, the tumors were allowed to progress until volumes reached approximately 100 mm^3^. After that, 70% of tumor in each mouse was resection to establish the postoperative recurrence model. To evaluate the photothermal therapeutic potential of MN systems, four distinct formulations including MN_B_@M_B_, MN_DOX_@M_B_, MN_B_@M_ICG_, and MN_DOX_@M_ICG_ were topically administered to the residual tumor tissue. Following a standardized 5‐min NIR laser irradiation, thermal response images of mice were quantitatively recorded using infrared thermal imager. Subsequently, the experimental processes were randomly divided into seven treatment groups (5 mice/group) including saline, free DOX, MN_DOX_@M_B_ without irradiation, MN_B_@M_ICG_ without irradiation, MN_DOX_@M_ICG_ without irradiation, MN_B_@M_ICG_ with irradiation, and MN_DOX_@M_ICG_ with irradiation. During the treatment processes, the body weight and tumor volume of each mouse were monitored every 2 days. At the end of the experiments, primary organs and tumors were obtained and underwent standardized fixation in 4% paraformaldehyde solution and subsequent microtome sectioning at 5 μm thickness for H&E, TUNEL, and Ki67 histological analyses.

### 4.9 Statistical Analysis

Quantitative results are presented as the mean ± standard deviation throughout the study. Statistical significance was calculated via unpaired Student's t‐tests. *p < 0.05, **p < 0.01, ***p < 0.001.

## Author Contributions

Y.W., Q.Z., and H.J. conceived the conceptualization and designed the experiment, G.K. and Q.Z. carried out the experiments and analyzed the data, G.K., W.L., Q.Z., and Y.W. wrote the paper.

## Ethics Statement

The animal experiments were approved by the Animal Ethics Committee of the Wenzhou Institute, University of Chinese Academy of Sciences (approval WIUCAS24090303), and complied with the recommendations of the academy's animal research guidelines.

## Conflicts of Interest

The authors declare no conflicts of interest.

## Supporting information


Supporting Information S1

## Data Availability

The data that support the findings of this study are available from the corresponding author upon reasonable request.
