# Supplementary material for: Hierarchical Cascade‐Activated Microneedle Patches for Photothermal and Chemotherapy
Source: Smart Med. 2026 Sep 30;5(5):e70059. doi: 10.1002/smmd.70059 (PMC13627266; doi:10.1002/smmd.70059)
Supplement: Supplementary file 1 [file SMMD-5-e70059-s001.docx]

**Supplementary**

**Hierarchical Cascade-Activated Microneedle Patches for Photothermal and Chemotherapy**

Gaizhen Kuang^1^, Wenzhao Li^1^, Yu Wang^1,^*, Qingfei Zhang^1,3,^*, Hua Jiang^1,2,^*

^1^ Department of Radiation and Medical Oncology, Joint Centre of Translational Medicine-WIUCAS, The First Affiliated Hospital of Wenzhou Medical University, Wenzhou, 325035, China

^2^ Department of Otolaryngology, The Second Affiliated Hospital, Zhejiang University School of Medicine, Zhejiang, China

^3^ School of Basic Medical Sciences, Wenzhou Medical University, Wenzhou 325000, China

Email: yuwang@wiucas.ac.cn (Y. Wang); zhangqf91@163.com (Q.F. Zhang); zrjh@zju.edu.cn (H. Jiang)

**Figure S1.** Microfluidic electrospray device for the fabrication of microspheres.

**Figure S2.** The photograph of M_B_ and M_ICG_ in tubes.

**Figure S3.** The ^1^H NMR spectra of (2E)-TCO-PNB (A) and DOX (B).

**Figure S4.** (A) The synthesis process of TCO-DOX. (B) The ^1^H NMR spectra of TCO-DOX.

**Figure S5.** The absorption spectra of TCO-DOX (A) and Tz-ICG (B).

**Figure S6.** The IEDDA bioorthogonal reaction of Tz-ICG and TCO-DOX.

**Figure S7.** The cell viability of B16F10 cells after a 24-hour incubation with Tz-ICG.

**Figure S8.** The body weight changes of tumor-bearing mice during the treatment processes of treatment.
